# Seeking to understand lived experiences of personal recovery in personality disorder in community and forensic settings – a qualitative methods investigation

**DOI:** 10.1186/s12888-017-1442-8

**Published:** 2017-08-01

**Authors:** Andrew Shepherd, Caroline Sanders, Jenny Shaw

**Affiliations:** University of Manchester, Manchester, UK

**Keywords:** Personal recovery, Personality disorder, Qualitative research

## Abstract

**Background:**

Understandings of personal recovery have emerged as an alternative framework to traditional ideas of clinical progression, or symptom remission, in clinical practice. Most research in this field has focussed on the experience of individuals suffering with psychotic disorders and little research has been conducted to explore the experience of individuals with a personality disorder diagnosis, despite the high prevalence of such difficulties. The nature of the personality disorder diagnosis, together with high prevalence rates in forensic settings, renders the understanding of recovery in these contexts particularly problematic. The current study seeks to map out pertinent themes relating to the recovery process in personality disorder as described by individuals accessing care in either community or forensic settings.

**Methods:**

Individual qualitative interviews were utilised to explore the lived experience of those receiving a personality disorder diagnosis and accessing mental health care in either community or forensic settings. A thematic analysis was conducted to identify shared concepts and understanding between participants.

**Results:**

Fourty-one individual participant interviews were conducted across forensic and community settings. Recovery was presented by participants as a developing negotiated understanding of the self, together with looked for change and hope in the future. Four specific themes emerged in relation to this process: 1. Understanding early lived experience as informing sense of self 2. Developing emotional control 3. Diagnosis as linking understanding and hope for change 4. The role of mental health services.

**Conclusions:**

Through considering personal recovery in personality disorder as a negotiated understanding between the individual, their social networks and professionals this study illustrates the complexity of working through such a process. Clarity of understanding in this area is essential to avoid developing resistance in the recovery process. Understanding of recovery in a variety of diagnostic categories and social settings is essential if a truly recovery orientated mental health service is to be developed.

## Background

Personal recovery is increasingly recognised as a principle goal for mental health services [1]. Understanding in this area is by its very nature idiographic, however efforts have been made to synthesise pertinent themes into framework conceptualisations [2] and to develop measures through which recovery orientated clinical practice may be enacted [3, 4]. So far, most research into the recovery process has been conducted with individuals with psychosis and the application of this developed understanding to the experience of individuals with other diagnoses requires further exploration.

Conceptualisation of personal recovery in relation to the experience of personality disorder is complex, given the underlying proposed nature of these diagnoses affecting areas of emotional regulation, the formation of personal relationships and maintenance of social roles [5], which can be seen as interacting directly with many of the domains outlined by Leamy, Bird et al. [2]. Despite this complexity a recent systematic review identified only three qualitative methods studies specifically focussed on the experience of recovery in relation to these diagnoses [6], in contrast to 89 studies identified through systematic review in relation to recovery in schizophrenia [7]. Understanding the recovery process with regard to personality disorder is further complicated by the high prevalence of the diagnoses within prison and other forensic settings, where approaching two-thirds of men and half of women are proposed as having a diagnosable personality disorder [8], in comparison with estimated rates of between one in 20 and one in six in the general community [9]. Within forensic settings particular issues and tensions can be seen as arising in relation to issues such as autonomy and empowerment that are crucial to understanding the process of recovery [10, 11]. While recovery focussed frameworks have been developed for care provision within forensic settings [12] there has been little exploration of the theoretical underpinning, or lived experience, of this process [13].

Research into the recovery process is essential in order that therapeutic support needs can be recognised and appropriately met through structured interventions [14]. Research can also facilitate the development of shared understanding between clinicians and patients - a necessary step if new interventions are to become standard for clinical services [15].

Therefore, with this background framework, the current study aimed to better map the lived experience of those receiving a personality disorder diagnosis, focussing on their understanding of personal recovery and the experiences of individuals accessing mental health care in either community (general community mental health and hospital) or forensic (prison and secure hospital) settings. Comparison is made between the experience of individuals accessing support in relation to their recovery in both settings, in order to consider in greater detail the particular complexities discussed above.

## Methods

Findings are drawn from a doctoral research project supported by funding from the National Institute for Health Research, registered with the UK Clinical Research Network (Reference 15,934). A qualitative methodological approach was adopted to adequately address the aims of the project; qualitative methods studies offer the opportunity for in-depth exploration of the personal aspects of health experience and illness narratives [16, 17]. Individual interviews were conducted with mental health service user participants - with participants initially identified based on their having received a personality disorder diagnosis. Subsequent rounds of recruitment were conducted in a purposive manner to address emergent themes and varying experiences of clinical care in different settings (forensic versus community).

The research was conducted in community and forensic clinical settings in the North of England – specifically within community hospitals, community mental health team bases, local prisons (Category B and Women’s prison), probation approved accommodation settings and regional secure hospitals. Participant anonymity is protected through anonymization, including the removal of any personal, or geographically identifiable information from interview transcripts.

### Individual interviews: - participant recruitment and interview process

Participants were selected on the basis of their having been identified as having received a personality disorder diagnosis and having sufficient spoken English language skill to enable them to participate in the interview process.

Potential participants were initially identified through approaching clinical teams with information relating to the study. Teams were asked to identify potential participants, and to provide them with information describing the role of participants in the study. Initial contact with participants by the researcher was therefore mediated through clinical teams.

No specific steps were taken to verify the personality disorder diagnosis and no specific subtype of diagnosis were sought. For the purpose of this research project it is argued that, on the basis of recent discussions of personality disorder diagnostic criteria [18–20] and possible future changes [9, 21–23], the present administration of any specific diagnosis is uncertain. Specifically, the recent revision of the DSM-5, from the previous edition, proposed a ‘hybrid’ system of personality disorder classification (combining categorical and dimensional considerations), that was ultimately proposed as needing further research; while the proposed changes for the next edition of the World Health Organisation’s diagnostic criteria (ICD-11) have suggested that most categorical distinctions (emotionally unstable versus dissocial personality disorder for example) be dropped in favour of an overarching personality disorder diagnosis, classified according to the severity of accompanying impairment [9]. Therefore, a pragmatic approach to diagnosis was adopted with no specific exclusion criteria set. A recruitment strategy was utilised where participants were being supported by their clinical team ‘as if’ they had a personality disorder diagnosis, and that the participants themselves identified with the diagnosis, as indicated through their consent to participate in the project. Participants were not excluded because of any co-morbid diagnoses. Therefore, no specific exclusion criteria were applied, excepting that participants were required to be able to offer informed consent for participation.

Participant recruitment was conducted in two waves; the first concentrating on community mental health settings and the second with a focus on potential participants with experience of accessing care within forensic settings. During the forensic recruitment wave a focus on individuals with experience of prison incarceration was developed such that all participants in the second wave had experience of prison incarceration in common. While only four participants were interviewed specifically in secure hospital settings the majority of forensic participants also had experience of hospital care within secure settings.

Interview recruitment and analysis were conducted in a parallel and iterative fashion; such that recruitment was informed in a theoretical manner. For example, early references to adverse inpatient experiences and the importance of consistent therapeutic contact led to participants being identified with varying lengths of contact with the mental health services and experience of contact in a variety of clinical settings (inpatient open ward, psychiatric intensive care unit, community care, prison and secure hospital). Recruitment continued until data saturation had been reached. The applied definition of saturation is discussed in the analysis section below.

As described above, potential participants were approached with information relating to the study by members of their clinical team. After expressing interest participants were then contacted by the first author and an appointment was arranged for the interview to be conducted. Interviews were conducted at clinical locations with which the participants expressed familiarity and comfort in attending. Prior to commencing the interview proper further opportunity was provided for participants to ask questions relating to the research. Consent for participation in the study was then obtained with a consent form being signed at this stage - although the consent process was viewed as being dynamic in nature, continuing throughout the period of the interview and beyond. Consent and interview were both undertaken at the same appointment to minimise disruption for participants.

Interviews were conducted in an open style, with initial questioning conducted in a fashion that encouraged the elaboration of personal story [24]. Semi-structured interview schedules were developed but were used only for participants who indicated they desired more prompting to elicit their experience. Specific topics identified for discussion within the interview schedules included: The experience of mental distress, first contact with mental health services, treatment and support accessed, understandings of treatment goals, conceptualisations of recovery, sources of support, experience of change since contact with services, and hope for change in the future. Interviews were audio-recorded and then stored, electronically, in an encrypted file format in keeping with NHS data protection standards.

### Analysis

Analysis was theoretically informed by a contextual constructivist approach to knowledge generation [25]. In this manner responses to questions were taken as representative of the participants’ understanding, but with consideration being given to the emergence of discourse as being a co-constructed phenomenon between researcher and participant.

The first step in the analysis process began with the writing of reflexive journal entries following each individual interview meeting. Journal entries allowed the capturing of significant themes based on initial reflection on the interview such that these could be explored in more detail during subsequent interviews, and during subsequent analysis steps. These initial themes were developed through reflection on subsequent interviews and further transformed throughout the analysis process. Data saturation was defined by the emergence of no novel themes within these journal entries over the course of sequential interviews.

Transcription of interviews was completed by the first author and represented the second phase in the analysis process, allowing an ‘immersion’ in the data [26]. The third step in the analytic process involved a coding strategy conducted in a manner so as to ‘fragment’ the transcribed data allowing horizontal comparison between interviews [27]. Memo-writing [28] was used to capture descriptions and links between coding and to allow the development of emergent themes [29]. Data analysis was supported using qualitative data analysis software (NVivo - QSR International version 11). The fourth stage of the analysis process involved the construction of thematic maps [30], which allowed the relationship between themes to be reviewed. Descriptive writing was then also incorporated into the analysis process.

Four randomly selected transcripts were coded by the second and third authors in a process of comparison and to prompt additional discussion. Any disagreement between coding, or in the interpretation of themes, was resolved through discussion and agreement during research meetings; as no disagreement emerged in relation to the underlying coding process research only a small minority of transcripts were coded by all authors to allow a greater focus on the elaboration and discussion of overarching themes. Themes were also discussed at meetings with a mental health service user advisory group throughout the research project. In this way analysis was reviewed from a variety of standpoints regarding theoretical experience and role. Issues of reflexivity, that is the impact of the role of the researcher on the research process [31], were also discussed during supervision and advisory group meetings in order to allow that they be sufficiently addressed.

### Ethical approval

Ethical approval was sought from the National Research Ethics Service East of England - Essex (Reference [14]/EE/0029). Access to prisons was approved by the National Offender Management Service, National Research Committee (Reference 2013–282); specific Prison Governor approval was granted for prisons from which participants were recruited. Access to hospitals and community mental health team bases was negotiated through NHS Trusts.

## Results

A total of 41 individual interview participants were recruited. Most participants self-identified as having been diagnosed with an Emotionally Unstable, or Borderline Personality Disorder, with some (principally in forensic settings) also reporting a diagnosis of Dissocial Personality Disorder. A minority of participants indicated that, while they agreed to participate in the study and had received a diagnosis of personality disorder they disagreed with the diagnosis. Demographic details of interview participants, together with the location of the interview are summarised in Table 1. The length of contact between the participant and mental health services ranged from less than one to 43 years (average length 12 years). The length of contact was roughly equal between community and forensic participants. Most community participants were not currently working at the time of their participation; examples of previous employment included armed forces service, manual labour, retail managerial positions, and healthcare. In describing their ethnic background all but five participants self-identified as White. The other five did not comment on this question. Individual interviews lasted between 15 and 79 min (mean 53 min, standard deviation 16 min).

**Table 1:** Participant codes and description

Code	Interview Setting	Age (as range)	Gender
*Int001*	Secure hospital ward	41–50	Male
*Int002*	Secure hospital ward	22–30	Female
*Int003*	Prison	41–50	Male
*Int004*	Prison	31–40	Male
*Int005*	Prison	22–30	Male
*Int006*	Prison	41–50	Male
*Int007*	Prison	31–40	Female
*Int008*	Prison	18–21	Male
*Int009*	Prison	18–21	Male
*Int010*	Prison	31–40	Male
*Int011*	Prison	41–50	Female
*Int012*	Prison	31–40	Female
*Int013*	Secure hospital ward	41–50	Male
*Int014*	Secure hospital ward	31–40	Male
*Int015*	General Community	31–40	Male
*Int016*	Prison	18–21	Female
*Int017*	Prison	31–40	Female
*Int018*	Prison	22–30	Female
*Int019*	Prison	22–30	Female
*Int020*	Prison	51–60	Female
*Int021*	Prison	41–50	Female
*Int022*	General Community	31–40	Male
*Int023*	General Community	41–50	Male
*Int024*	General Community	31–40	Female
*Int025*	General Community	18–21	Female
*Int026*	General Community	51–60	Female
*Int027*	Community inpatient ward	51–60	Male
*Int028*	Community inpatient ward	41–50	Female
*Int029*	General Community	21–30	Female
*Int030*	General Community	31–40	Female
*Int031*	General Community	41–50	Female
*Int032*	General Community	41–50	Female
*Int033*	General Community	31–40	Male
*Int034*	Community inpatient ward	22–30	Male
*Int035*	General Community	41–50	Female
*Int036*	General Community	22–30	Female
*Int037*	Community inpatient ward	41–50	Male
*Int038*	General Community	41–50	Female
*Int039*	General Community	18–21	Female
*Int040*	General Community	31–40	Female
*Int041*	General Community	18–21	Male

In discussing their understanding of recovery, participants described an overarching process involving a balance between developing an ‘understanding of self’ together with ‘looked for change’ or hope for the future; this process was not simply an individual act however but involved a close negotiation of understanding between the individual, their host social network and other agents, such as professionals, with whom they developed contact. Within this overarching process four specific themes emerged as representative of the work undertaken in ‘recovery’: 1. Understanding early lived experience as informing sense of self 2. Developing emotional control 3. Diagnosis as linking understanding and hope for change 4. The role of mental health services. Each of these four themes is explored in greater detail below; illustrative quotations are used for the richness of their description and, where possible, to represent counter-arguments or statements. The number of participants endorsing specific themes is not presented below, in keeping with the qualitative epistemology, however words are employed to imply quantity at times in the following fashion; many (approximately 75% or more), most (more than 50%), minority (less than 50%).

Understanding early lived experience as informing sense of self.

Most participants framed their understanding of their experiences within a description of their early life within their family, particularly their sense of belonging and the interpretations of their behaviour made by key family members.

“I always felt there was a lot of pressure on me to do very well, because my brothers are both very bright and had done well at school and I always felt compared to them [brothers]when I went to primary school..”

Later in the interview this respondent reflected on her current sense of self:

“It’s difficult because I sometimes feel like my illness has kind of defined who I am, I’m just like the one who’s got all the problems and I’ve not really found who I am yet.” [Int036].

For other participants, early life experience was characterised by a sense of alienation from their family, leading to them struggling to develop a sense of their own ‘place’ within the social unit:

“I had a lot of depression and down days, when I think back now just not fitting in even the foods that I liked were totally different I had nothing in common with the family that I lived with and brought up with. Not in the food, nothing.” [Int038].

Within the context provided by their social networks, participants saw some elements of behaviour as constituting a destructive aspect of themselves. These elements were conceptualised as emerging in response to experiences of violence and pain, with many participants referring to early experiences of emotional and physical abuse. This impacted on their ability to trust in others and form relationships:

“I won’t let many people in, I choose my circles… who I speak to even smaller… I still choose not to speak to a lot of people about it. Just mainly because I kind of deal with it, or I’ve dealt with it and I don't feel like bringing it up.” [Int019].

Participant accounts of their experience were therefore intimately framed within the understanding of their social networks, often reaching back to early life experiences of family life - accounts which were often coloured by experiences of violence or abuse within the family environment.

### Developing emotional regulation

Many participants, when discussing hoped for change, described their wish for greater control over their emotional life, as a process of developing a more coherent understanding of their experience. This then became an intimate part of the ‘recovery process’ - a greater sense of stability, or ‘self-control’:

“I think in terms of, like, recovery, in terms of being able to have a degree of self-control and being able to think ahead about the consequences of things so that rather than having a big blow up.” [Int033].

Participants engaging in acts of self-harm, or suicidal behaviours, positioned these as emerging directly from experiences of trauma, or distress, and representing a potential relief from conflict; linking their emotional distress to a sense of embodiment – that is, they developed an explicit link between ‘somatic’ and ‘mental’ understandings of distress and pain [32, 33]:

*Interviewer*: “What type of things lead to you feeling you need space”.

*Participant*: “because my emotions go up and down where I’m angry and then really really mad, then I feel suicidal it’s like a volcano with me. At the moment I’m like level but let anything change tonight and it goes.”

*Interviewer*: “What makes it change, what type of things set off the volcano?”

*Participant*: “It’s when I don’t feel safe and stuff I just don’t feel like I can do it no-more and basically at the end of the day, it’s just like, like I said before, I just wish I was dead, because it would stop all the arguing with everybody.” [Int040].

### Diagnosis as linking understanding and hope for change

For most participants, the application of a personality disorder diagnosis represented an important step in the understanding of their experience. An appreciation of diagnosis allowed them to begin a process of engagement and to develop a sense of hope for the future:

“That helped knowing a little bit and then I didn't really get a lot of support with regards to what I had, I did an awful lot of research myself […] But then by having that that opened up other avenues, other courses of treatment and having regular CPN [Community Psychiatric Nurse] was great, really but it was good to be diagnosed with something anyway, because I knew it was something worse [than depression].” [Int023].

“They gave me the diagnosis of emotionally unstable personality disorder. So I was put on, obviously, several antipsychotic drugs and antidepressants which were linked with an anti-anxiety as well and I started going to a hearing voices group, which was near where I lived, so that made things a lot easier knowing that I was with like-minded people.” [Int025].

For a minority of participants however the diagnosis of personality disorder was seen as unhelpful - representing a direct comment on them as a person, or as a representation of their previous behaviour, not a ‘mental illness’ per se:

*Participant*: “It felt like a bit of an attack to me own, everything about me, you know, everything that I am do you know?”

*Interviewer*: “That your personality is who you are?”

*Participant*: “Yeah” [Int003].

“Well the doctor said I’ve got an antisocial personality disorder, I’m not antisocial so where do they get that from? […] Well technically, that could be right I suppose, you know, it’s like antisocial burgling and crime, stuff like that isn't it but you know does every Tom, Dick and Harry who’s in [prison] now have an antisocial personality disorder just because they’re in?” [Int013].

This understanding was particularly pertinent in prison settings where diagnosis was seen as being used, through expert witness testimony, to inform the judicial process, or as a means of excluding some from care within a hospital setting.

For another group of participants, the recovery process was seen as being one of radical change, representing an adaption in self-understanding beyond that offered within a diagnostic framework:

“I changed quite a lot to be fair, I pretty much did become a completely different person. […] I gained empathy, I gained compassion, I gained understanding these were things that were lacking, even before my mental health problems really, they were just accentuated with my mental health problems.” [Int023].

### The role of the mental health services

Relationships with professionals in a therapeutic setting were seen as being crucial in allowing the individual an opportunity to reflect on experience and plan for future change:

*Participant*: “The counsellor that I saw was the best person.”

*Interviewer*: “What was best about the counsellor, what was it about them?”

*Participant*: “We had a great rapport.”

*Interviewer*: “So the relationship with the counsellor was important to you?”

*Participant*: “Yeah, very important, and I trusted her […] It let me open up more to her and to know that she cared, and she really did care, and she was very interested in me and my thoughts…” [Int025].

Others described how their relationships with professionals had been dismissive, or even bullying, in nature:

“I felt hang on I feel more bipolar, than I do, with that symptom included, and I look back how I was as a kid, because sometimes I get quite hyper. I tried hanging myself at 14 as well so I was suicidal from a young age and I don’t know it just fits more. He [*psychiatrist*] said it was so I could get out of going under this team at [*region*] […] which I’ve been fighting not to go under ‘cause [*social worker*] I don’t get on with him, I don’t find him useful, I find him patronising and not at all good, and I’m not the only one with that opinion so he was saying I was just doing it, saying it so I wouldn’t, didn’t have to go under them and I wasn’t” [Int040].

“But within the illness it’s difficult for me to understand it I just try and go along I got the understanding that people don't trust it or they say it’s a cop out. But I don't care about it I know I’m ill I know the things I’ve done, I know I wouldn't be in this service if there was nothing wrong with me” [Int015].

Within prison settings participants reflected on the role of prison officers and their interaction with prisoners during times of mental distress. Prison officers were seen as representing the front line of support in some cases, but also as not appreciating the complexity of distress that they witnessed:

“…the prison officers and that were pretty good with me, because they knew I was mentally unwell. So even though I was locked in my room, because if you don't go to workshops and stuff in prison you get what’s called basic salary and you don't get near normal, amounts, but because they knew I was unwell they gave me enhanced and they gave me a television even though I wasn't going to workshops and they took me out each day to get me a shower and at exercise times, so they were quite good to me…” [Int001].

“I understand that they’re not emotionally connected to me, they don't really give a shit, it’s a job - everything. Well to some extent they do, they’ve got a duty of care, you know, if I died tonight I’d be forgot in a week, do you know what I mean, it’s all it really is nobody gives a shit in here…” [Int003].

## Discussion

The present study sought to explore the experience and personal meaning of recovery in relation to individuals receiving a personality disorder diagnosis and with experience of accessing care in either community or prison settings. Overall, the process was revealed as a negotiation of understanding between those experiencing mental distress, their social networks and clinical (or other) professionals. In keeping with previous research, recovery was identified not as a discrete outcome but instead as an on-going process [34]. The way this process was understood and reflected was determined largely by the individual’s sense of themselves and their reflection on their lived experience. Social networks, as in other studies, were seen as playing an essential role in this ‘sense-making’ activity [2, 6, 13].

Differences in this process emerged between those participants with experience of care solely in the community and those with experience of incarceration in prison. For all participants, the process involved a process of ‘fitting’ the diagnosis alongside their sense of themselves in their personal identity. For those who were, or had been, classified as offenders however this process could be seen as being still more complex, as they contended with feelings of ‘double’ stigmatisation – making sense of being an ‘offender’, ‘mentally ill’ or ‘personality disordered’ [35]. Concepts of mental disorder therefore became incorporated into other understandings of self, for example ideas of rehabilitation [36], or ‘redemption’ [37, 38]; for some the idea of ‘personality disorder’ was helpful for this process, for others it was not - and was rejected.

Diagnosis, for the majority, represented a route through which understanding of past distress could be linked to current experience, although this was not a universal understanding with other participants viewing the diagnosis as inherently stigmatising or as leading to an exclusion from health service support, a finding consistent with other studies comparing the experience of those receiving a personality disorder diagnosis with other forms of mental disorder [39]. In keeping with this, as stated above, a minority of participants elected to participate in the study – stating that they had received a personality disorder diagnosis but were rejecting of the classification. This difficulty was perhaps particularly noteworthy when considered in the context of forensic healthcare settings where a few participants experienced the diagnosis of personality disorder as being used to exclude them from care options, such as hospital transfer. Despite these difficulties when considered in the light of individual experience many found the act of diagnosis to be a powerful act allowing an alternative perspective to be adopted and hope for future change to develop.

Mental health services were seen as supportive in their ability to offer therapeutic relationships that allowed participants to work through their understanding of recovery in a negotiated manner. However, the capacity to develop these relationships was being impinged upon by tensions between modes of sense making - with many participants detecting uncertainty from clinical staff in terms of their understanding of the diagnosis of personality disorder; such uncertainty impacted on the individual’s ability to foster feelings of hope in relation to change. Within prison settings other professionals, principally prison officers, were seen as fulfilling an essential role in the support of those with experience of mental distress. The impact of this emotional labour on officers can-not be directly commented on from the findings in this study, although - given the described impact of such work on clinical professionals - it can be hypothesised that this will represent a significant burden. Caution is necessary to ensure that the well-recognised difficulties of working with individuals with disrupted attachment experience [40], as is often characteristic of forms of personality disorder, does not lead to a process of exclusion for ‘difficult patients’ [41].

Overall, a close articulation can be seen between the construct of recovery as a developing understanding of self, as described by participants here, with concepts such as ‘Connectedness’ and ‘Identity’ [2] or ‘Existential Recovery’ [42]. It is also apparent that ‘recovery’ should be conceptualised as occurring within a ‘social space’ involving work not just by individuals but also by their social networks and mental health care services [43]. In this manner, the current findings serve to strengthen the role of existing recovery frameworks in terms of their applicability to individuals receiving a diagnosis of personality disorder, while also illustrating the challenges for this process in different clinical settings and diagnoses – that is the complexity of moral understanding inherent in ‘offender recovery’ and the challenge around clarity of understanding in relation to the nature of the diagnosis of personality.

### Strengths and limitations

Systematic review has revealed the limited amount of research conducted in relation to the concept of recovery in personality disorder; what research has been conducted has generally focussed on the experience of participants accessing care within community settings. By focussing on the experience of individuals across a variety of setting this study builds on, and adds to, this previous knowledge and understanding.

Reflexivity represents the manner in which the researcher teams’ own theoretical experiences and understandings interact with the analysis of the available material [31, 44]. All interviews and much of the analysis process for this study were undertaken by the first author; at the time a higher trainee in forensic psychiatry and doctoral research fellow. The author’s role as a psychiatrist was known to all participants in the study and may have impacted on the emergent discourse [45]. This impact was considered during research supervisory meetings with the remaining authors together with coding approaches and emergent themes. Themes were also discussed and developed through meetings with a service-user advisory group recruited at the outset of the project. In this manner interpretation of findings was considered in a multi-disciplinary fashion, acknowledging the impact of the researcher role on the investigation and moving to prevent a one-sided reading of the data [46].

The majority of participants within the present study self-identified as White; this is significant as it is known that race and ethnicity are factors that influence the understanding of personality disorder diagnoses [47, 48]. Additionally it is recognised that cultural heritage may produce different appreciations of the recovery process [2, 4]. A decision was taken in this study not to focus on race or ethnicity within the purposive sampling strategy: - on the basis of the complexities outlined further research is required specifically focussing on the experience of race in relation to personality disorder and personal recovery and with particular attention paid to issues of reflexivity.

### Future work

A significant theme emerging from this study is the way understandings of recovery are negotiated between the individual with experience of mental distress, their social networks and clinical staff or other professionals – emphasising the importance of similar claims made in other settings [49]. An intimate sensitivity to the language used is apparent in this process and further understanding relating to the dynamic nature of this process is required. Studies focussing on the development of dialogue and discourse between agents are therefore required to explore and map this process.

As discussed above the experience of Black and Ethnic Minority individuals with a personality disorder diagnosis need further exploration - studies should be developed to capture this missing experience in an in-depth fashion.

Finally, the role of prison officers in supporting individuals experiencing mental distress within prison settings was also highlighted. Further research should be undertaken to explore the nature of this process in greater detail - focussing particularly on the impact of such emotional labour on officers and the availability of appropriate support, or supervision, to allow this role to be fulfilled.

## Conclusion

The recovery process, in relation to the experience of those diagnosed with a personality disorder, was revealed to be one of developing self-understanding in relation to one’s biographical experience - with an emerging sense of greater control in relation to emotional experience. This understanding involved negotiation between the individual and their host social networks, as well as clinical professionals and other agents providing support. This negotiation proved particularly complex for those faced with the work of also coming to terms with classification as having ‘offended’ against society, adding a moral dimension to the process. For some however this process was seen as being disrupted by the varying attitudes of clinical staff that were at times perceived as being almost hostile in their manner, an experience that was seen as particular to the diagnosis of personality disorder. The understanding and support for the process of personal recovery in relation to mental disorder is complicated by varying understandings of its implications among professionals [50, 51].

The findings from the current study highlight the potential difficulty in the development of a negotiated understanding between clinical professionals and individuals who receive a personality disorder diagnosis. Emergent tensions in relation to the understanding and communication of diagnosis further complicate this process. A lack of clarity in this area risks the development of stigmatised narratives leading to a sense of exclusion and hopelessness. The central role of social networks in the recovery process also requires attention from mental health services; this may represent a problem for those offering care within forensic settings where individuals may be divorced, or separated by great distances, from original networks.

Research into the process and meaning of personal recovery is crucial for the continuing development of clinical mental health services. This understanding may be particularly complex in the case of personality disorder. The current study highlights the importance of attention to communication and collaboration between professional and patient to allow the development of mutual understanding. Developing understanding of recovery in a variety of diagnostic categories and social settings is essential if a truly recovery orientated mental health service is to be developed.
